# Patients’ experience and satisfaction towards virtual health care during the COVID-19 pandemic in southern region of Saudi Arabia

**DOI:** 10.1097/MD.0000000000041443

**Published:** 2025-02-07

**Authors:** Abdulaziz M. Al-Garni, Ayed A. Shati, Hasan S. Alamri, Syed E. Mahmood, Awad S. Alsamghan

**Affiliations:** a Department of Internal Medicine, Psychiatry Division, College of Medicine, King Khalid University, Abha, Saudi Arabia; b Department of Child Health, College of Medicine, King Khalid University, Abha, Saudi Arabia; c Department of Family and Community Medicine, College of Medicine, King Khalid University, Abha, Saudi Arabia.

**Keywords:** COVID-19, pandemic, patient satisfaction, virtual health care

## Abstract

Health care providers can use these virtual platforms for delivering medical advice and prescriptions to patients. This study was aimed to explore the patients’ experiences and level of satisfaction regarding virtual health care received during the coronavirus disease-19 (COVID-19) pandemic. This study also assessed the before and during lockdown sleep quality in these participants. The current study included 522 participants from Saudi Arabia. Virtual health care satisfaction questionnaire was implemented to record the data on patient experience toward the virtual health care during COVID-19 pandemic. Patients expressed a high level of satisfaction with virtual health care services during the COVID-19 pandemic, as indicated by a mean score of 4.15 on a five-point Likert scale, which translates to an 83% satisfaction rate. Most participants felt they could communicate effectively with their doctors, appreciated the good picture and sound quality of their virtual appointments, and felt that their privacy was respected. Additionally, they reported comfort during history taking and examinations, and felt that doctors adequately explained solutions, including prescriptions and advice. Interestingly, the study found no significant association between the type of specialty or patient demographic factors and the level of satisfaction. In terms of sleep quality, a comparison of subjective sleep parameters before and during the lockdown revealed significant changes. The results from a *t* test indicated that mean scores for various sleep components: such as sleep duration, sleep disturbances, sleep latency, daytime dysfunction, habitual sleep efficiency, and subjective sleep quality, showed significant differences (*P* < .001) between the 2 assessment periods. Overall, the mean scores for these components increased, indicating a deterioration in sleep quality during the lockdown period. The study found that most participants were satisfied with the virtual health care system, noting its benefits in reducing overcrowding, care delays, and unnecessary in-person visits during pandemics. This increased accessibility could enhance patient satisfaction and lower costs, though it may not completely replace traditional hospital visits. The researchers recommend further studies with a larger, more diverse group to better understand patient experiences and improve telehealth services in Saudi Arabia. Overall, while the virtual system shows promise, more research is needed to optimize its use.

## 1. Introduction

In face of an exponential increase in cases of coronavirus disease-19 (COVID-19), health care systems worldwide are racing to adopt virtual consultations and treatment approaches.^[1]^ This obviates the need for physical meetings between patients and health care providers, therefore increasing the safety for patients as well as for health care providers. The virtual or digital health care is cost effective and secure use of data and communication technologies to deal with several medical conditions without seeing the patient face-to-face.^[2]^ Health care providers can use different virtual platforms such as real-time audio–video meeting or audio/text messaging to address patient’s concerns. Health care providers can use these virtual platforms for delivering medical advice and prescriptions to patients.^[1,3]^ The use of virtual health care has been suggested as a method to maintain a continuum of health care for patients.^[4,5]^ As a result, the prevalence of virtual care has rapidly increased during this COVID-19 pandemic. There has been exponential increase in virtual health care consultations in the United States, China, Canada, UK, Italy, Germany, India, and many African countries during this COVID-19.^[1]^ In 2020, one of the study conducted in USA reported that the virtual consultations for telemedicine increased within 4 weeks from <1% to 70%.^[6]^ In addition to these findings, several studies documented that virtual health care system along with telemedicine contribute to various health-related aspects such as to reduce crowding in hospitals and clinics, preventing nonemergency cases from unnecessary exposure to COVID-19 zones, and providing safety for both patients and health care providers.^[2,7–9]^ However, a lower percentage of physicians and patients are adequately trained to use these digital services.^[2]^ Other major concerns are the common problems in connectivity and how much the patients are satisfied with this virtual health care system. Albarrak AI and colleagues reported that the main problems associated with virtual consultations and telemedicine was patient’s privacy, high equipment cost, inadequate training and lack of connection between information technology experts and clinicians.^[10]^

With regard to patient’s level of satisfaction for virtual health care system, 1 retrospective cohort study conducted in New York reported that patient satisfaction with video visits was high.^[11]^ Overall 72% of elderly patients were satisfied with telemedicine for home health services in another study. Authors reported that telemedicine significantly reduced the clinic visits.^[12]^

In 2017, the telehealth application called “Seha” was launched in Saudi Arabia. This application is used all over the kingdom for virtual medical consultation through voice and video calls. Since the surge of COVID-19 cases started, the Ministry of Health of Saudi Arabia suspended clinical visit, canceled nonemergency appointments and encouraged the community to use digital applications either for counseling or to attend appointments remotely in order to control the spread of disease. Another study reported that patients were highly satisfied for virtual consultation during COVID-19. The level of satisfaction was 80.4%.^[13]^ This study has a sample size limitation. However, overall there is a scarcity of related data that can signify the patient’s satisfaction level for the virtual health care system.

Above discussed literature signifies the importance of virtual health care system during this COVID-19 pandemic. But there are some important concerns such as the level of satisfaction of patients for this virtual health care system. There is scarcity of such reports from the region of Saudi Arabia. Further studies are required to assess patient perceptions of receiving virtual medical care, its advantages and challenges during the ongoing pandemic. Thus, current study was aimed to evaluate the patient experience toward the virtual health care during the COVID-19 pandemic in Saudi Arabia.

## 2. Design and methods

### 2.1. Study design and participants

The current study is an observational descriptive cross-sectional study. The aim of the study was to explore patients’ opinions regarding the virtual health care received during the COVID-19 pandemic, to associate the type of specialized medical services received and patient socio-demographic factors with patients’ level of satisfaction towards the virtual health care received, to explore the factors associated with dissatisfaction among patients during the virtual health care sessions, and to compare the before lock down and during lock down sleep quality to explore the impact of COVID-19 pandemic. The study was conducted from August 29, 2020 to November 29, 2020. Initially we recruited 583 participants from Saudi Arabia who have used virtual health care platforms at least once during the pandemic and aged ≥ 18 years. Among these participants, 61 participants were dropped out due to missing data, health-related issues, lack of technical ability, and currently not residing in Saudi Arabia. Finally, 522 participants were included in this study. We hypothesized that the patients prefer and are satisfied regarding virtual health care during the COVID-19 pandemic in Saudi Arabia. The research question was: What are the patients’ experiences and satisfaction regarding virtual health care during the COVID-19 pandemic in Saudi Arabia?

### 2.2. Participant inclusion criteria

Participants should have access to required technology and the internet, is comfortable with using audio/video technology, has no physical/sensory/cognitive disabilities that would limit audio/video conference use, has access to email/chat, and is comfortable with using the modality.

### 2.3. Assessment of patient experience toward the virtual health care

Virtual health care satisfaction questionnaire was implemented to record the data on patient experience toward the virtual health care. The questionnaire’s purpose is to evaluate the satisfaction and viewpoints of patients regarding digital health care during COVID-19. This questionnaire contains questions regarding socio-demographic characteristics (Table 1) and telemedicine satisfaction (Table S1, Supplemental Digital Content, http://links.lww.com/MD/O346).

**Table 1:** Demographics characteristics of the participants (n = 522).

Age	
18–25 years	291 (55.7)
26–35 years	105 (20.1)
36–45 years	74 (14.2)
46–55 years	35 (6.7)
Above 55 years	17 (3.3)
Gender	
Male	201 (38.5)
Female	321 (61.5)
Educational level	
Primary school	1 (0.2)
Middle school	9 (1.7)
High school	92 (17.6)
University	370 (70.9)
Postgraduate studies	50 (9.6)
Nationality	
Saudi	503 (96.4)
Non-Saudi	19 (3.6)
Residency	
Central Saudi Arabia	156 (29.9)
Eastern Saudi Arabia	38 (7.3)
Western Saudi Arabia	98 (18.8)
Northern Saudi Arabia	22 (4.2)
Southern Saudi Arabia	208 (39.8)

All values are presented as numbers and percentages.

### 2.4. Ethical approval

Current study was approved (Approval No.: ECM#2020-1002) by the Research Ethics Committee, King Khalid University, Saudi Arabia on 19/08/2020. All participants provided their oral and written consent. Participants were assured that the information will be handled anonymously and for scientific purposes only.

### 2.5. Statistical analysis

The data analysis related to evaluation of patient experience toward the virtual health care included 2 stages. The first stage included a descriptive analysis, in which numerical variables were reported in terms of means, standard deviations and standard errors. The second stage included hypothesis testing using the Pearson Chi-square test and Likert scale analysis. The five-point Likert scale is a widely used method for measuring attitudes or opinions by assigning numerical values to various levels of agreement. This approach enables respondents to convey their feelings along a continuum, which simplifies the analysis and interpretation of the data. Moreover, we employed paired samples *t* test to compare the responses of participants for the before lock down and during lock down sleep quality. All the data was analyzed in SPSS (Version 22.0).

## 3. Results

### 3.1. Descriptive analysis

#### 3.1.1. Socio-demographic information

Socio-demographic information of all the participants is presented in Table 1. Among the participants, 291 (55.7%) belonged to younger age group of 18 to 25 years and 80.5% of total participants had received university level education. Majority of the participants (n = 503; 96.4%) included in the current study are Saudi nationals.

#### 3.1.2. Patients’ opinions regarding virtual health care and Likert scale analysis

Patients’ opinions regarding virtual health care received during the COVID-19 pandemic era are summarized in Table S1, Supplemental Digital Content, http://links.lww.com/MD/O346. The “five-point Likert scale” gives weight as value 4.21 to 5.00 (Strongly Agree), 3.41 to 4.20 (Agree), 2.61 to 3.40 (Neutral), 1.81 to 2.60 (Disagree), and 1.00 to 1.80 (Strongly Disagree) is presented in Table 2. Results showed that most participants agreed that they were able to communicate adequately with doctors (189 (36.2%) strongly agreed and 212 (40.6%) agreed), the picture and sound quality of the virtual appointment were good (203 (38.9) strongly agreed and 214 (41%) agreed), their privacy was respected during the consultation (267 (51.1) strongly agreed and 196 (37.5) agreed), they were comfortable during history taking and exams that were done (210 (40.2) strongly agreed and 218 (41.8) agreed), and the doctor explained solutions including prescribing medicine and/or providing advice (222 (42.5) strongly agreed and 196 (37.5) agreed). The mean and standard deviations of all the 5 statements are presented in Table 3. Overall, statement 3 (my privacy was respected during consultations) has the highest satisfaction level of 4.36 (Strongly agree). The mean score of all the 5 statements is 4.15, reflecting 83% level of satisfaction of participants towards virtual health care (Table 3).

**Table 2:** Patients’ level of satisfaction towards the virtual health care received (5-point Likert scale analysis).

S. no.	Statement	Strongly agree n (%)	Agree n (%)	Neutral n (%)	Disagree n (%)	Strongly disagree n (%)
1.	I was able to communicate adequately with my doctor	189 (36.2)	212 (40.6)	85 (16.3)	24 (4.6)	12 (2.3)
2.	The picture and sound quality were good	203 (38.9)	214 (41)	78 (14.9)	17 (3.3)	10 (1.9)
3.	My privacy was respected during consultations	267 (51.1)	196 (37.5)	45 (8.6)	7 (1.3)	7 (1.3)
4.	I was comfortable during history taking and exams that were done	210 (40.2)	218 (41.8)	65 (12.5)	15 (2.9)	14 (2.7)
5.	The doctor explained to me solutions including prescribing medicine and/or providing advice	222 (42.5)	196 (37.5)	63 (12.1)	22 (4.2)	19 (3.6)

**Table 3:** Patients’ level of satisfaction towards the virtual health care received (5-point Likert scale analysis).

S. no.	Statement	Mean	SD	Rank	Level of satisfaction
1	I was able to communicate adequately with the doctor	4.04	0.96	5	Agree
2	The picture and sound quality were good	4.12	0.91	3	Agree
3	My privacy was respected during consultations	4.36	0.80	1	Strongly Agree
4	I was comfortable during history taking and exams that were done	4.14	0.93	2	Agree
5	The doctor explained to me solutions including prescribing medicine and/or providing advice	4.11	1.02	4	Agree
Mean score		4.15			

### 3.2. Bivariate analysis

#### 3.2.1. Testing the association between the types of specialized medical services received with the patients’ level of satisfaction towards the virtual health care received (Chi Square test)

Table 4 present the association between the types of specialized medical services received and the patient’s level of satisfaction towards the virtual health care (reported using the Pearson Chi Square test). Results showed no significant association between the type of specialized medical services received and the patient’s level of satisfaction towards the virtual health care.

**Table 4:** Testing the association between the types of specialized medical services received with the patients’ level of satisfaction towards the virtual health care received (Chi Square test).

Specialty attended	Level of satisfaction					*P*-value
	Strongly Agree n (%)	Agree n (%)	Neutral n (%)	Disagree n (%)	Strongly Disagree n (%)	
Internal medicine	34 (6.51)	49 (9.38)	9 (1.72)	5 (0.95)	2 (0.38)	.223
General surgery	10 (1.91)	11 (2.10)	0 (0)	0 (0)	0 (0)	
Obstetrics and gynecology	8 (1.53)	5 (0.95)	2 (0.38)	0 (0)	0 (0)	
Pediatrics	15 (2.87)	17 (3.25)	2 (0.38)	0 (0)	0 (0)	
Family medicine	33 (6.32)	26 (4.98)	5 (0.95)	2 (0.38)	2 (0.38)	
Orthopedic	8 (1.53)	9 (1.72)	2 (0.38)	2 (0.38)	0 (0)	
Dental	12 (2.29)	12 (2.29)	1 (0.19)	1 (0.19)	1 (0.19)	
Psychiatry	8 (1.53)	3 (0.57)	0 (0)	1 (0.19)	1 (0.19)	
General	51 (9.77)	50 (9.57)	13 (2.49)	0 (0)	7 (1.34)	
Ophthalmology	14	11 (2.10)	2 (0.38)	0 (0)	0 (0)	
Others	32 (6.13)	30 (5.74)	13 (2.49)	0 (0)	1 (0.19)	

No significant association was found between type of specialized medical services received and patient’s level of satisfaction at 0.05 level of significance.

#### 3.2.2. Testing the association between socio-demographic factors with patients’ level of satisfaction towards the virtual health care received (Chi Square test)

Table 5 presents the association between patient’s demographic factors and their level of satisfaction towards the virtual health care (reported using the Pearson Chi Square). No significant association was found between the demographic factors and patient level of satisfaction towards the virtual health care.

**Table 5:** Testing the association between socio-demographic factors with patients’ level of satisfaction towards the virtual health care received (Chi Square test).

Demographics	Level of satisfaction					*P*-value
	Strongly Agree n (%)	Agree n (%)	Neutral n (%)	Disagree n (%)	Strongly Disagree n (%)	
Age						
18–25 years	129 (24.71)	126 (24.13)	26 (4.98)	3 (0.57)	7 (1.34)	.159
26–35 years	40 (7.66)	46 (8.81)	12 (2.29)	5 (0.95)	2 (0.38)	
36–45 years	31 (5.93)	33 (6.32)	6 (1.14)	2 (0.38)	2 (0.38)	
46–55 years	12 (2.29)	14 (2.68)	5 (0.95)	1 (0.19)	3 (0.57)	
Above 55 years	13 (2.49)	4 (0.76)	0 (0)	0 (0)	0 (0)	
Gender						
Male	74 (14.17)	93 (17.81)	21 (4.02)	5 (0.95)	8 (1.53)	.157
Female	151 (28.92)	130 (24.90)	28 (5.36)	6 (1.14)	6 (1.14)	
Educational level						
Primary school	1 (0.19)	0 (0)	0 (0)	0 (0)	0 (0)	.095
Middle school	2 (0.38)	3 (0.57)	2 (0.38)	0 (0)	2 (0.38)	
High school	46 (8.81)	39 (7.47)	5 (0.95)	1 (0.19)	1 (0.19)	
University	153 (29.31)	161 (30.84)	39 (7.47)	8 (1.53)	9 (1.72)	
Postgraduate studies	23 (4.40)	20 (3.83)	3 (0.57)	2 (0.38)	2 (0.38)	
Nationality						
Saudi	215 (41.18)	217 (41.57)	47 (9.00)	10 (1.91)	14 (2.68)	0.649
Non-Saudi	10 (1.91)	6 (1.14)	2 (0.38)	1 (0.19)	0 (0)	

No association found between all demographic factors and patient level of satisfaction at the 0.05 level of significance.

#### 3.2.3. Exploring the factors associated with dissatisfaction among patients during the virtual health care sessions

We also explored the dissatisfaction among patients during their virtual health care sessions (Table 6). The dissatisfaction includes problem related with internet connection, taking long time and difficulty in explaining different concern.

**Table 6:** Exploring the factors associated with dissatisfaction among patients during the virtual health care sessions.

Problems encountered	Overall experience satisfaction				
	Very satisfied n (%)	Satisfied n (%)	Neutral n (%)	Dissatisfied n (%)	Very dissatisfied n (%)
Internet connection	16 (3.06)	37 (7.08)	30 (5.74)	5 (0.95)	10 (1.91)
Difficulty in using the platform	7 (1.34)	16 (3.06)	13 (2.49)	3 (0.57)	5 (0.95)
Long time until start	32 (6.13)	54 (10.34)	66 (12.64)	5 (0.95)	10 (1.91)
Difficulty in clarifying my concerns	27 (5.17)	42 (8.04)	45 (8.62)	9 (1.72)	5 (0.95)
Health care provider was uncooperative	0 (0)	2 (0.38)	14 (2.68)	2 (0.38)	6 (1.14)
None	107 (20.49)	74 (14.17)	20 (3.83)	0 (0)	4 (0.76)

### 3.3. Analyses of the sleep quality before and during COVID-19 lock down

#### 3.3.1. Frequency distribution of participants with good and bad sleep before and during COVID-19 pandemic lock down

Frequency distribution of good and bad sleep sleepers was computed based on the PSQI global scores obtained at the 2 assessment levels, that is, before and during the COVID-19 pandemic lock down. Before lockdown scores showed that 87% participants were good sleepers (i.e., PSQI < 5) and 13% were bad sleepers (i.e., PSQI ≥ 5), whereas during lock down assessment showed that the distribution of bad sleepers increased significantly increased (*P* < .001) from 13% to 83% and the distribution of good sleepers significantly reduced (*P* < .001) from 87% to 17%.

#### 3.3.2. Subjective sleep parameters

Results of *t* test showed that the mean scores of all the components namely sleep duration (*P* < .001); sleep disturbances (*P* < .001), sleep latency (*P* < .001), daytime dysfunction (*P* < .001), habitual sleep efficiency (*P* < .001), and subjective sleep quality (*P* < .001) significantly differed between 2 assessment levels. The mean scores for these components increased, indicating a decline in sleep quality during the COVID-19 pandemic lockdown.

## 4. Discussion

The novel COVID-19 pandemic has altered several aspects such as economy, society, and health care system. Health care systems worldwide are racing to adopt virtualized consultations and treatment approaches.^[1]^ Telehealth programs such as virtualized consultations and treatment approaches overcome physical barriers to provide patients and caregivers access to convenient medical care.^[6]^ As a result, the prevalence of virtual care has rapidly increased thought out the world during this COVID-19 pandemic.^[1]^ However, there are certain concerns regarding the use of virtual health care system, reflecting the need of an assessment of level of satisfaction of people for these virtual interface platforms.^[2]^ Authors report that inadequate training and lack of connection between information technology experts and clinicians and less privacy were the main problems associated with virtual consultations and telemedicine.^[10]^

Despite of the facts, a previous study conducted in Saudi Arabia, evaluated the physician’s willingness towards adopting telemedicine in clinical practice.^[10]^ Most physicians showed positive perceptions towards virtual health care and were willing to adopt it in clinical practice. Issues related to privacy, training, cost, information and communication technology for the adoption of virtual health care system need to be addressed.

One study was conducted in British Columbia, Canada regarding the satisfaction of patients about virtual visits and patient centered care.^[14]^ Nearly 93.2% respondents stated their virtual visits were of high quality, 91.2% participants reported them helpful to resolve their health issue, and 79% were confident in their security and privacy. This is somewhat in line with findings of our current study, where responses of majority of the participants reflect that they were comfortable and satisfied with virtual health care. These findings together support for the adaptation of virtual health care system in the clinical settings.

In current study the level of satisfaction towards virtual health care was high (83%). This echoes the finding of previous study published about patient satisfaction toward a virtual health care in endocrinology clinics in Saudi Arabia.^[13]^ Authors reported that the level of satisfaction for virtual health care system was about 80.4%. A study was conducted in Chile aimed to assess patient satisfaction with tele-neurology and it was found that the level of satisfaction was high about 97%.^[15]^ One study evaluated usability and satisfaction of telemedicine for head and neck ambulatory visits.^[16]^ This study reported that the patients were highly satisfied with telemedicine. Another study was aimed to characterized telemedicine and correlates it with patient’s level of satisfaction.^[17]^ This study showed that the overall 85% of patients rated their satisfaction with their telemedicine physician 5 stars on scales of 0 to 5. About the problems participants faced during virtual session, long waited time was on top followed by difficult to clarify their concerns and internet connection problems. Our findings support AlJaloud and colleagues observation where nearly half of the Saudi participants wanted to continue the use of virtual services during the post COVID-19 pandemic era.^[18]^ Saudi Arabian citizens believe that telemedicine saves time, labor and costs and is an effective tool to treat coronavirus patients at a safe distance.^[19]^

Our findings are also comparable to the Argentina study which created and implemented a virtual care program for patients with respiratory tract infections during the epidemiological outbreak.^[20]^ They deemed this new care strategy could prevent hospital overcrowding, reduce long delays, avoid unnecessary referrals, and limit infections in waiting rooms. The inability to meet the health-care professional face-to-face was reported by 53.8% patients experiencing virtual consultation in primary health care centers in Riyadh.^[21]^

Telemedicine promotes social distancing and delaying of nonurgent medical consultations and surgeries in a pandemic situation.^[22]^ The virtual clinic services in Saudi Arabia provide health-care consultations in different fields of medicine and public health. However, the exact number of virtual clinics is unreported.^[21]^ The Sehhaty mobile application can be used to book an appointment at the health care facility. The “Seha Virtual Hospital” provides specialized virtual services.^[23]^ Other applications (TETAMMAN, TABAUD, and TAWAKKALNA) that have been launched have improved the quality of using telemedicine activities and facilitated all health services.^[24]^ This digital health innovation has a huge potential for increasing continuous patient engagement with health clinics.^[25]^

Specific patient education experiences can be helpful for patient learning about virtual care. Community-based knowledge sharing is also encouraged.^[26]^ Our sample size had less number of older people, the probable reason is they were less likely to have used or familiar telehealth. The results of an Australian population support this need for better support for older people to access remote modes of care.^[27]^ Willingness to use telemedicine in future was high in the recipients as well as the health care providers during COVID-19 pandemic in UK.^[28]^ The findings from a Saudi study showed that 70.6% of participants were aware of the existence of virtual clinics and one-third of the participants had virtual consultations during the post-pandemic era.^[29]^ Over 90% Saudis preferred having virtual appointments through telephone and video calls using hospital-provided platforms rather than in-person visits according to a recent study.^[30]^ The satisfaction measurement factors of telemedicine may change in the future. Du, Y. et al developed a meaningful tool for future studies for evaluating patient satisfaction with telemedicine. This scale involves 9 dimensions, such as humanistic care, doctor-patient communication, service efficiency, diagnosis and treatment result, ease of use, system quality, usefulness, privacy and security, overall satisfaction.^[31]^ In our study, demographic factors or specialty variations didn’t have a major effect on satisfaction levels. This implies that satisfaction might be more affected by aspects like the quality of care, communication, or the overall patient experience, rather than demographic details or the specific medical specialty. For health care providers, this suggests that enhancing the overall patient experience and care quality could be more beneficial than customizing services based on demographic or specialty variations.

The limitation of this study is the implicit bias usually present in this descriptive, online voluntary response survey, and the relatively low response rate. The dropout rate of participants raises concerns about sample representativeness and potential bias. Moreover, the study’s reliance on voluntary participation introduces self-selection bias, as individuals who are more satisfied or dissatisfied with virtual health care may be more inclined to participate. The inclusion criteria focus primarily on technological access and comfort rather than broader factors that could influence patient satisfaction, such as socio-economic status or health literacy. This narrow focus may limit the generalizability of the findings. Other study limitation is a small sample size and limitation of generalizability of the results due to the small sample size. We hope in the future to have all the required resources to do multicentric/nationwide studies with a larger sample size. However, as the importance of patient satisfaction continues to increase in the provision of health care, this work is considered to be an important step in this direction, particularly in Saudi Arabia.

## 5. Conclusions

Findings from the current study reflected that most of the participants were satisfied with virtual health care system. The virtual health care could help prevent overcrowding in health facilities especially in such pandemics, decrease delays in attention and avoid unnecessary in-person visits, easing access to health care services possibly by providing patients with needed care at appropriate time. All of these could have a significant impact on costs and patient satisfaction but not necessarily displacing in-person visits to hospital and clinics. Future studies with a larger sample size and multicentric in nature need to further explore the patient’s experiences and level of satisfaction regarding virtual health care in Saudi Arabia. This will help to further continue and expand the telehealth services in the Kingdom. It will also give telemedicine providers insights into areas where they can improve their services.

## Acknowledgments

The authors extend their appreciation to the Deanship of Scientific Research at King Khalid University for funding this work through large group Research Project under grant number: RGP2/378/44.

## Author contributions

**Conceptualization:** Abdulaziz M. Al-Garni, Ayed A. Shati.

**Formal analysis:** Syed E. Mahmood.

**Investigation:** Abdulaziz M. Al-Garni, Ayed A. Shati, Hasan S. Alamri.

**Methodology:** Hasan S. Alamri, Awad S. Alsamghan.

**Validation:** Awad S. Alsamghan.

**Visualization:** Awad S. Alsamghan.

**Writing – original draft:** Ayed A. Shati, Syed E. Mahmood.

**Writing – review & editing:** Syed E. Mahmood.

## Supplementary Material


