# Supplementary material for: Patients’ experience and satisfaction towards virtual health care during the COVID-19 pandemic in southern region of Saudi Arabia
Source: Medicine (Baltimore). 2025 Feb 7;104(6):e41443. doi: 10.1097/MD.0000000000041443 (PMC11813006; doi:10.1097/MD.0000000000041443)
Supplement: Supplementary file 1 [file medi-104-e41443-s001.docx]

| **Supplementary Table 1. Patients’ opinions regarding virtual health care received during the COVID-19 pandemic era** | |
| --- | --- |
| Questions | Respondents n (%) |
| Who was attending the virtual clinic? (n=522) |  |
| Me | 351 (67.2) |
| Child under my care | 56 (10.7) |
| Older person under my care | 115 (22) |
| How was the virtual clinic conducted? (n=522) |  |
| Sound only | 465 (89.1) |
| Video | 57 (10.9) |
| What was the reason for using a virtual clinic? (n=522) |  |
| Appointment | 95 (18.2) |
| Counseling and medical advice | 349 (66.9) |
| Emergency | 78 (14.9) |
| With what medical specialty did you meet? (n=522) |  |
| Internal medicine | 99 (19) |
| General surgery | 21 (4) |
| Obstetrics and gynecology | 15 (2.9) |
| Pediatrics | 34 (6.5) |
| Family medicine | 68 (13) |
| Orthopedic | 21 (4) |
| Dental | 27 (5.2) |
| Psychiatry | 13 (2.5) |
| General | 121 (23.2) |
| Ophthalmology | 27 (5.2) |
| Others | 76 (14.6) |
| What is your opinion on receiving virtual health care in comparison to traditional, in-person, medical visits? (n=522) |  |
| Better than traditional | 179 (34.3) |
| As good as traditional | 168 (32.2) |
| Worse than traditional | 56 (10.7) |
| Not sure | 119 (22.8) |
| Do you prefer to attend your next appointment/counseling in a non-virtual clinic? (n=522) |  |
| Yes | 236 (45.2) |
| No | 86 (16.5) |
| Maybe | 200 (38.3) |
| Do you prefer to keep using telemedicine platforms after the pandemic ends? (n=522) |  |
| Yes | 217 (41.6) |
| No | 103 (19.7) |
| Maybe | 202 (38.7) |
| Do you feel that the physical presence of a doctor and a physical examination by him/her may give you more trust in the diagnosis / treatment? (n=522) |  |
| Yes | 367 (70.3) |
| No | 35 (6.7) |
| Maybe | 120 (23) |
| Do you think your presence in the clinical health care unit is essential for your confidentiality in treatment?(n=522) |  |
| Yes | 261 (50) |
| No | 108 (20.7) |
| Maybe | 153 (29.3) |
| What problems did you face during these sessions? (n=522) (r=606)* |  |
| Internet connection | 98 (18.8) |
| Difficulty in using the platform | 44 (8.4) |
| Long time until start | 167 (32) |
| Difficulty clarifying my concerns | 128 (24.5) |
| Healthcare provider was uncooperative | 24 (4.6) |
| None | 205 (39.3) |
| Do you think virtual health care helps to reduce the risk of exposure to COVID-19 virus? (n=522) |  |
| Yes | 396 (75.9) |
| No | 30 (5.7) |
| Maybe | 96 (18.4) |
| Overall experience satisfaction(n=522) |  |
| very dissatisfied | 22 (4.2) |
| dissatisfied | 13 (2.5) |
| neutral | 117 (22.4) |
| satisfied | 194 (37.2) |
| very satisfied | 176 (33.7) |
| All values are presented as numbers and percentages.  *n = sample size or total number of cases, r = total number of responses in multiple choices | |
